# Muscle depletion in cirrhotic patients assessed using computed tomography: a cross-sectional study

**DOI:** 10.1590/1516-3180.2019.0436.R1.19122019

**Published:** 2020-04-22

**Authors:** Elisa Sfoggia Romagna, Marcelo Campos Appel-da-Silva, Eiji Suwa, Fabio Kunihiro Maeda, Angelo Alves de Mattos, Cristiane Valle Tovo

**Affiliations:** I MD. Endocrinologist and Postgraduate Student, Department of Hepatology, Universidade Federal de Ciências da Saúde de Porto Alegre (UFCSPA), Porto Alegre (RS), Brazil.; II MD. Gastroenterologist and Postgraduate Student, Department of Hepatology, Universidade Federal de Ciências da Saúde de Porto Alegre (UFCSPA), Porto Alegre (RS), Brazil.; III MD. Gastroenterologist, Hospital Nossa Senhora da Conceição (HNSC); and Radiologist, Imaging Service, Unimed Porto Alegre, Porto Alegre (RS), Brazil.; IV Physicist, Nuclear Medicine Service, Santa Casa de Misericórdia de Porto Alegre, Porto Alegre (RS), Brazil.; V MD. Physician, Department of Hepatology, Universidade Federal de Ciências da Saúde de Porto Alegre (UFCSPA), Porto Alegre (RS), Brazil.; VI MD, PhD. Adjunct Professor and Coordinator, Postgraduate Program on Hepatology, Universidade Federal de Ciências da Saúde de Porto Alegre (UFCSPA), Porto Alegre (RS), Brazil.

**Keywords:** Liver cirrhosis, Sarcopenia, Mortality, Malnutrition., Muscle depletion, Cirrhosis, Chronic liver disease.

## Abstract

**BACKGROUND:**

Sarcopenia is a common complication in patients with cirrhosis and may lead to increased morbidity and mortality.

**OBJECTIVE:**

To investigate the prevalence of sarcopenia and its association with disease severity scores, among patients with cirrhosis.

**DESIGN AND SETTING:**

Observational and retrospective cohort study carried out in a tertiary-care hospital in southern Brazil.

**METHODS:**

This study was conducted among patients with chronic liver disease who were followed up at the gastroenterology and hepatology outpatient clinic of a tertiary-care hospital in southern Brazil and who underwent computed tomography scans of the abdomen through any indication.

**RESULTS:**

We included 83 patients in the study. In the population evaluated, there was a predominance of males (57.80%) and the mean age was 56 years. Hepatitis B or C virus was present in the genesis of the disease in 34.9% of the cases, followed by an etiology of alcohol abuse (30.1%). Sarcopenia was diagnosed in 41 (49.4%) of the patients when the cutoff point for cirrhotic patients was used. There was no significant correlation between the Child-Pugh and MELD severity scores and the occurrence of sarcopenia.

**CONCLUSION:**

Sarcopenia presents high prevalence among patients with chronic liver disease, without any association with predictors of severity.

## INTRODUCTION

Chronic liver disease is a major global public health problem. Liver cirrhosis and hepatocellular carcinoma respectively account for over 1.2 million and 800,000 deaths annually.^1^^,^^2^^,^^3^^,^^4^^,^^5^ According to a study conducted in Brazil, liver diseases are the eighth leading cause of death among patients treated through the public healthcare system, and cirrhosis is the most prevalent type.^6^

Protein-energy malnutrition is frequently observed in cases of liver cirrhosis. This, in association with low physical activity, may result in sarcopenia. The prevalence of protein-energy malnutrition is around 20%-30% among patients with chronic liver disease and over 60% among patients with advanced cirrhosis.^7^^,^^8^^,^^9^^,^^10^^,^^11^

Sarcopenia has been described as a syndrome characterized by progressive and extensive loss of strength and skeletal muscle mass, with a risk of unfavorable outcomes, including patient morbidity and mortality.^7^^,^^11^^,^^12^^,^^13^^,^^14^ It is one of the most common complications in cirrhotic patients, with prevalence ranging from 30% to 70%, and it involves reduced quality of life and increased infection rates. It is an independent mortality factor that implies a worse outcome after liver transplantation.^11^^,^^15^^,^^16^^,^^17^^,^^18^ However, the diagnostic criteria are not uniform.

The European Working Group on Sarcopenia in Older People (EWGSOP) has defined a list of diagnostic criteria. In its latest publication (2018), this group recommended that both low muscle mass and low muscle function (strength or performance) should be used to diagnose sarcopenia.^12^^,^^19^ The justification for using two criteria is based on the fact that muscle strength does not depend on muscle mass alone.^12^^,^^13^^,^^19^^,^^20^ Therefore, when only the ‘muscle mass’ is evaluated, the term ‘muscle depletion’ can be used.

On the other hand, the European Association for the Study of the Liver (EASL) recommends that, among cirrhotic patients, sarcopenia should be evaluated by means of abdominal computed tomography (CT). This parameter has been validated using dual-energy x-ray emission densitometry, which is considered to be the gold standard.^11^ However, there is divergence in the literature regarding the best cutoff point for diagnosing sarcopenia in patients with cirrhosis, using CT.^21^^,^^22^

## OBJECTIVE

The aims of this study were to investigate the prevalence of sarcopenia among cirrhosis patients and to ascertain whether there might be an association between sarcopenia and disease severity.

## METHODS

A cross-sectional study was conducted using data obtained through a review of medical records. The patients included were aged 18 years or older, had been diagnosed with cirrhosis of any etiology, had undergone abdominal CT through any indication and were being followed up at the gastroenterology and hepatology outpatient clinic of a tertiary-care hospital in Porto Alegre, southern Brazil. The vast majority of the CT scans were performed to evaluate the presence of liver lesions, but CT was also indicated for evaluating abdominal pain in patients attended at the emergency unit, and also within the routine evaluation for liver transplantation.

The exclusion criteria consisted of occurrences of cases of HIV co-infection, organ transplantation and inadequate records.

Cirrhosis was diagnosed from the clinical findings, laboratory tests, imaging examinations and/or upper digestive endoscopy, and from histopathological examinations.

The review of the medical records was based on data that had been obtained at the time when the abdominal CT scan was performed. The variables analyzed were age, sex, etiology of liver disease, Child-Pugh score,^23^ model of end-stage liver disease (MELD) score,^24^ ascites at the time of CT, previous history of hepatic encephalopathy, previous history of digestive bleeding, presence of hepatocellular carcinoma (HCC) and patient outcome (death or liver transplantation).

**Figure 1.**
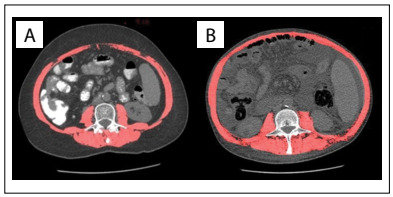
Cross-sectional area of third lumbar vertebra (L3) on abdominal computed tomography in a patient with sarcopenia (32.17 cm²/m²) (A) and a patient without sarcopenia (52.3 cm²/m²) (B).

Sarcopenia was diagnosed by means of abdominal CT scans. On these, the third lumbar vertebra (L3) was identified and the transverse area of the abdominal and paraspinal wall muscles involved (psoas, erector spinae, quadratus lumborum, transversus abdominis, internal and external oblique muscles and rectus abdominis) was measured (Figure 1). This measurement in square centimeters was divided by the patient’s height squared, and is referred to as the L3 skeletal muscle index (L3 SMI). The muscle area in this region is commonly used for diagnostic purposes because it includes central skeletal muscles whose mass is independent of activity and water retention, and it corresponds best to the patient’s total muscle mass. All the CT images were analyzed by the same medical physicist using the ImageJ software, which is similar in accuracy to other types of software currently used in diagnosing sarcopenia.^25^

Two pairs of cutoff points for L3 SMI were used for further comparisons. The first was based on Carey et al.,^26^ who evaluated a specific cutoff point for patients with cirrhosis, and defined that sarcopenia was present when L3 SMI < 50 cm²/m² in men and < 39 cm²/m² in women. The second was based on Prado et al,^27^ who evaluated patients with solid tumors of the respiratory and gastrointestinal tract and took cutoff points for sarcopenia of < 52.4 cm²/m² for men and < 38.5 cm²/m² for women. This second pair of cutoff points are the ones used by the EWGSOP.^12^^,^^19^

For the statistical analysis, categorical variables were described according to the frequency and percentage. The sarcopenia prevalence rate was presented with its respective 95% confidence interval (95% CI). Normally-distributed quantitative variables were described in terms of the mean and standard deviation. Categorical variables were compared using the chi-square test or Fisher’s exact test. A 5% significance level was used for the comparisons.

The study was approved on November 8, 2011, by the ethics committee of the institution involved, under the protocol number 3675/11.

## RESULTS

Among the 570 cirrhotic patients seen at the outpatient clinic, 466 had not undergone abdominal CT, and there was no record of height for 21 patients. Thus, the final sample consisted of 83 patients. These were predominantly male patients (48; 57.80%), with a mean age of 56.68 ± 10.40 years (63.9% were younger than 60 years of age), and a mean body mass index (BMI) of 27.5 ± 4.3 kg/m^2^. Regarding etiology, 34.9% of the cases were due to hepatitis B or C, and 30.1% were due to alcohol abuse.

At the time of the evaluation, 40 patients (48.2%) had ascites; 29 (35.4%) had presented upper gastrointestinal bleeding in the past; and 28 (33.7%) had a history of hepatic encephalopathy. The other characteristics of the population are presented in Table 1. Eight of the patients (10%) died as a consequence of liver disease.

Sarcopenia was identified in 41 patients (49.4%; 95% CI 38.2%-60.6%) according to the cutoff point specific for cirrhotic patients; and in 40 patients (48.2%; 95% CI 37.1%-59.4%) according to the cutoff point used for oncological patients (P = 0.976).

**Table 1.** Clinical characteristics of the patients using the cirrhosis cutoff points (< 50 cm²/m² in men and < 39 cm²/m² in women)

	With sarcopenia	Without sarcopenia	P
	n = 41	n = 42	
ETIOLOGY, n (%)			0.383
Alcohol	23 (56.1)	19 (45.2)	
Other	18 (43.9)	23 (61.1)	
CHILD-PUGH, n (%)			0.383
A	22 (53.7)	18 (49.2)	
B and C	19 (46.3)	24 (57.1)	
MELD, n (%)			
> 15	7 (17.1)	14 (33.3)	0.147
≤ 15	34 (82.9)	28 (66.7)	0.147

MELD = model of end-stage liver disease.

Non-obese patients (BMI < 30 kg/m²) presented sarcopenia more frequently than obese patients, regardless of the cutoff point used (P = 0.012 and P = 0.017 respectively).

Among the patients with an etiology of alcohol abuse for cirrhosis, 23 (56.1%) had sarcopenia according to the cutoff point for cirrhotic patients and 23 (57.5%) according to the oncological cutoff point. Among patients with other etiologies, 18 cases (43.9%) of sarcopenia were identified using the cirrhosis cutoff point and 17 (42.5%) were identified using the oncological cutoff point; there was no difference between these etiology groups (P = 0.38 and P = 0.27 respectively).

Evaluation of the patients in terms of compensated cirrhosis (Child-Pugh A) and decompensated cirrhosis (Child-Pugh B and C) showed that among the 41 patients diagnosed with sarcopenia according to the cirrhosis-specific cutoff point, 22 (53.7%) were Child-Pugh A and 19 (46.3%) were Child-Pugh B and C (P = 0.383) (Table 1). Among the 40 patients diagnosed with sarcopenia according to the oncological cutoff point, 21 (52.5%) were Child-Pugh A and 19 (47.5%) were Child-Pugh B and C (P = 0.513) (Table 2).

**Table 2.** Clinical characteristics of the patients using the oncological cutoff points (< 52.4 cm²/m² in men and < 38.5 cm²/m² in women)

	With sarcopenia	Without sarcopenia	P
	n = 40	n = 43	
ETIOLOGY, n (%)			0.275
Alcohol	23 (57.5)	19 (44.2)	
Other	17 (42.5)	24 (55.8)	
CHILD-PUGH, n (%)			0.513
A	21 (52.5)	19 (44.2)	
B and C	19 (47.5)	24 (55.8)	
MELD, n (%)			
> 15	7 (17.5)	14 (32.6)	0.185
≤ 15	33 (82.5)	29 (67.4)	0.185

MELD = model of end-stage liver disease.

There was no significant association between MELD score and sarcopenia (r = -0.035; P = 0.147) using the cutoff of MELD > 15. There was no correlation between sarcopenia and Child-Pugh score according to the cirrhosis-specific cutoff point (P = 0.518) or the oncological cutoff point (P = 0.632) (Table 2).

There were no significant differences in the numbers of patients with upper gastrointestinal bleeding (P = 1.00), hepatic encephalopathy (P = 1.00) or ascites (P = 1.00) according to the cutoff point.

Age over 60 years was not significantly associated with either cutoff point. There were 18 patients (21.7%) with type 2 diabetes mellitus, and seven of these (17%) had sarcopenia, which was not significant. Twenty-six patients (31.3%) had HCC and, of these, eight (19.5%) had sarcopenia according to the two cut-off points (P = 0.040).

Among the eight patients who died, two had sarcopenia according to both cutoff points (25%; P = 0.275 for the cirrhosis cutoff point; and 25%; P = 0.269 for the oncological cutoff point).

## DISCUSSION

The nutritional status of patients with cirrhosis is increasingly stressed in the literature. In recognition of the impact of muscle loss in these patients, we conducted a study to assess the prevalence of sarcopenia and its relationship with disease severity, with comparisons between different cutoff points used for making the diagnosis. We found that the prevalence of sarcopenia was 49.4% when we used a cutoff point established specifically for cirrhotic patients. Since few studies have evaluated sarcopenia in cirrhotic patients, we also used a cutoff point that had previously been evaluated for an oncological population, which resulted in a prevalence rate of 48%. The results did not differ significantly between the two cutoff points.

The prevalence rate found in the present study is concordant with the findings of Jeong et al., who evaluated a similar outpatient population of 131 cirrhotic patients who underwent CT. Using a cutoff point established by Prado et al.,^27^ they found a prevalence of 48.9%.^28^

Tandon et al. also evaluated cirrhotic patients (most of them compensated) in outpatient follow-up with CT or magnetic resonance imaging, and found a prevalence of 43%.^29^ Among non-liver transplantation cirrhotic patients evaluated using CT, Hanai et al. found a higher prevalence of sarcopenia (68%).^17^

Interestingly, Montano-Loza et al.^30^ and Tandon et al.^31^ found lower prevalence of sarcopenia than what was observed in the present study (40% and 41%, respectively). However, they evaluated patients on the liver transplantation waiting list whose condition was more severe. Likewise, Meza-Junco et al. evaluated 116 patients on liver transplantation lists and found a prevalence of sarcopenia of 30%.^32^ On the other hand, Giusto et al. found a prevalence of 78% among patients who were eligible for liver transplantation.^18^ In Brazil, Zambrano et al. evaluated cirrhotic patients who were being followed up as outpatients and found a prevalence of 17%.^33^

There are divergences of opinion regarding which cutoff points should be used for diagnosing sarcopenia. Prado et al. evaluated patients with solid tumors of the respiratory and gastrointestinal tract and determined that cutoffs of 52.4 cm²/m² for men and 38.5 cm²/m² for women were associated with increased mortality.^27^ Jones et al. used this cut-off point among colorectal cancer patients, and Sheean et al. used it among patients with respiratory failure.^34^^,^^35^ This is also the cutoff point currently recommended by the EWGSOP.^12^^,^^19^

Among patients with liver cirrhosis, there is even greater disagreement about which cutoff point to use, and there have been changes over the years. Tandon et al.,^29^^,^^31^ Giusto et al.^18^ and Montano-Loza et al.^30^ evaluated the prevalence of sarcopenia among cirrhotic patients using the cutoffs proposed by Prado et al.^27^ However, Montano-Loza et al. subsequently proposed another cutoff point for diagnosing sarcopenia in cirrhotic patients: 50 cm²/m² for men and 42 cm²/m² for women.^36^^,^^37^ A new study by the same group, this time assessing the inclusion of sarcopenia in MELD, then used cutoffs based on Martin et al. (≤ 53 cm²/m² for men and ≤ 41 cm²/m² for women with BMI ≥ 25 kg/m², and ≤ 43 cm²/m² for all patients with BMI < 25 kg/m²).^38^^,^^39^ The most recent study on this topic, published by Carey et al. in 2017, was a multicenter study to determine cutoffs for diagnosing sarcopenia in cirrhotic patients. A total of 396 patients were assessed at five liver transplantation centers in the United States, and the cutoff point that was most significant for detecting survival differences between the groups was < 50 cm²/m² for men and < 39 cm²/m² for women.^26^

In view of the controversy regarding the best cutoff point for diagnosing sarcopenia, we chose to evaluate the ones that are most used, i.e. the cutoffs advocated by Carey et al., obtained from cirrhotic patients, and by Prado et al., obtained from patients with solid tumors. These were compared, and no significant difference in the prevalence of sarcopenia was found between them.

Regarding the etiology of cirrhosis, 34.9% of the cases were due to hepatitis B or C, which is a proportion similar to what has been described in the literature.^30^ However, there was no statistically significant difference regarding the prevalence of sarcopenia and etiology. Grouping patients according to whether the etiology of their cirrhosis was alcohol abuse did not result in any significant difference.

Regarding sarcopenia and predictors of mortality (Child-Pugh and MELD), some studies did not find any relationship between sarcopenia and the degree of hepatic dysfunction.^17^^,^^31^^,^^32^ However, others found a relationship and suggested that including sarcopenia assessment in Child-Pugh and MELD scores could improve mortality predictions among cirrhotic patients.^31^^,^^38^^,^^40^ In the present study, there was no correlation between sarcopenia and the Child-Pugh and MELD severity scores, although it should be pointed out that sarcopenia was not included in the MELD score as a prognostic tool.

Although it has been shown that sarcopenia increases mortality among cirrhotic patients,^37^^,^^41^^,^^42^ there was no association in the present study between mortality and sarcopenia, which might be explained by the fact that the sample consisted of outpatients, with disease of lower severity.

The main factor that may have contributed towards potential study limitations was the small sample size, which perhaps contributed to the lack of significant differences between severity, mortality and the different cutoff points. Also, the varied indications for CT were potentially a source of bias concerning clinical status. In addition, the retrospective design involved inherent limitations that should be taken into account. On the other hand, the situation depicted in this study represents the real life of outpatients in a reference center.

## CONCLUSION

The present study indicated that there was high prevalence of sarcopenia among individuals with cirrhosis, even if this was compensated. In addition, no difference between the cutoff points that were used to diagnose sarcopenia was found, and there was no association between the severity predictor scores (Child-Pugh and MELD) and presence of sarcopenia. Future studies with larger samples could contribute towards better understanding of this topic.
